# Type 1 diabetes complicated with cyclic vomiting syndrome and exogenous insulin antibody syndrome: A case report

**DOI:** 10.3389/fendo.2022.1043301

**Published:** 2022-11-10

**Authors:** Leiluo Geng, Xue Diao, Hao Han, Ying Lin, Wei Liang, Aimin Xu

**Affiliations:** ^1^ State Key Laboratory of Pharmaceutical Biotechnology, The University of Hong Kong, Hong Kong, Hong Kong SAR, China; ^2^ Department of Medicine, School of Clinical Medicine, The University of Hong Kong, Hong Kong, Hong Kong SAR, China; ^3^ Department of Endocrinology, The University of Hong Kong-Shenzhen Hospital, Shenzhen, China; ^4^ Department of Pharmacology and Pharmacy, The University of Hong Kong, Hong Kong, Hong Kong SAR, China

**Keywords:** type 1 diabetes, cyclic vomiting syndrome, exogenous insulin antibody syndrome, hypoglycaemia, case report

## Abstract

Every fifth individual with type 1 diabetes (T1D) suffers from an additional autoimmune disorder due to shared genetic factors and dysregulated immunity. Here we report an extremely rare case of T1D complicated with cyclic vomiting and hypoglycaemia. A 27-year-old Chinese woman with 14-year history of T1D was periodically hospitalized for severe vomiting of more than 30 times a day without apparent organic causes. The vomiting developed acutely and remitted spontaneously after 2-3 days, followed with intractable hypoglycaemia for another 3-4 days during the hospitalization. A few weeks after discharge, she was admitted once again with the same symptoms and disease course. Cyclic vomiting syndrome (CVS) was diagnosed according to the Rome IV criteria, a system developed to define the functional gastrointestinal disorders. Dynamic association and disassociation of exogenous insulin and insulin antibodies (IAs) were identified in her blood during hypoglycaemia, leading to the diagnosis of exogenous insulin antibody syndrome (EIAS). Treatment with rituximab to suppress the IAs was associated with a striking amelioration of hypoglycaemia. Unexpectedly, the episodes of cyclic vomiting were also dramatically reduced. In conclusion, we identified the first case with alternating CVS and EIAS in the setting of T1D. Dynamic measurements of free and total insulin are helpful for the diagnosis of EIAS. CVS is likely to be a latent autoimmune disorder considering the good response to rituximab treatment.

## Introduction

Type 1 diabetes (T1D) is an autoimmune disorder characterized by T-cell mediated autoimmune destruction of pancreatic β-cells in genetically predisposed individuals, eventually causing severe insulin deficiency and hyperglycaemia (1). Genetic susceptibility plays a crucial role in the development of T1D and more than 50 human leukocyte antigen (HLA) regions as well as non-HLA genes have been associated with T1D (1). As the pathogenesis of various autoimmune diseases share common genetic factors and immunologic processes, every fifth individual with T1D suffers from an additional autoimmune disorder, such as celiac disease, autoimmune thyroid disease, autoimmune gastritis, vitiligo, pernicious anemia and others (2). In general, female sex, older age, and longer duration of diabetes confer a greater risk of multiple autoimmune diseases (3). Some autoimmune diseases are underdiagnosed or missed in patients with T1D due to their similar symptoms to the diabetic complications. Accurate identification of concurrent autoimmune disorders in the setting of T1D is critical and essential for early-intervention and precise treatment.

Patients with T1D are vulnerable to diabetic gastroparesis and suffer from vomiting attacks due to increased tendency to synthesise ketone bodies and decreased gastrointestinal motility with autonomic neuropathy (4). However, T1D complicated with cyclic vomiting syndrome (CVS) is very rare. To date, there was only one case reported from Japan (5). CVS is an idiopathic functional vomiting disorder characterized by recurrent, stereotypical episodes of severe nausea, vomiting and abdominal pain interspersed with periods of little or no symptoms (6). The exact pathogenesis of CVS is unclear and there are no specific biomarkers for this disease. Some potential contributors to CVS have been summarized from previous cases, including psychological dysfunction, dysregulation of the brain-gut axis, mitochondrial DNA mutation, dysfunction of the endocannabinoid system, and overreaction of the hypothalamic-pituitary-adrenal (HPA) axis (6). The diagnostic criteria for adult CVS according to the Rome IV criteria are stereotypic episodes of vomiting with the following characteristics: at least two acute-onset episodes in the past 6 months, each occurring at least 1 week apart, and persisting for less than 1 week. Furthermore, there is an absence of vomiting between episodes, but other milder symptoms can occur between cycles. Supportive findings include a personal or family history of migraine (7).

Due to absolute reliance on exogenous insulin and defective glucose counterregulation, patients with T1D are susceptible to symptomatic hypoglycaemia (8). The common causes of hypoglycaemia in T1D include insulin overdose, irregular food intake, and improper physical activity (8). Although it is extremely rare, some patients with T1D generate autoantibodies against the insulin receptor (IRAb) (9), which induce hypoglycaemia *via* functioning as insulin receptor agonists and impairing normal insulin clearance (10, 11). Meanwhile, T1D patients receiving insulin therapy may generate antibodies against exogenous insulin or insulin analogs (12), causing dysglycaemia with intractable hypoglycaemia, which is named as exogenous insulin antibody syndrome (EIAS) (13). Standardized assays for IRAb or insulin antibodies (IAs) are not common in hospital laboratories, possibly leading to missed or underdiagnosed autoimmune hypoglycaemia in T1D.

Here we discuss an adult patient of T1D suffering from concurrent CVS and EIAS. Her symptoms of vomiting followed by spontaneous hypoglycaemia during the hospitalization were extremely rare and showed strong periodicity and insensitivity to multiple conventional treatments. Unexpectedly, rituximab (a chimeric monoclonal anti-CD20 antibody to deplete circulating B cells) was effective in alleviating the symptoms of both CVS and EIAS.

## Case report

**Figure 1:**
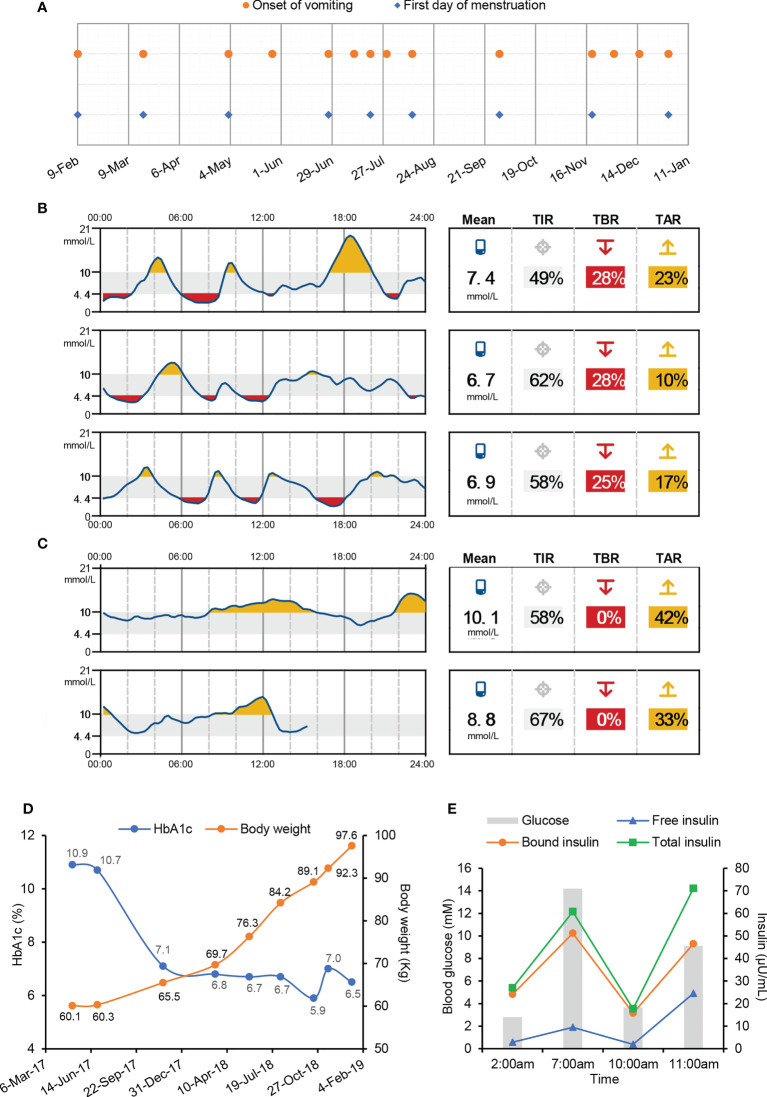
Symptoms of cyclic vomiting and intractable hypoglycaemia in this T1D patient. **(A)** Date of vomiting onset and the first day of menstruation. **(B, C)** Continuous blood glucose monitoring after the remission of vomiting **(B)** and before discharge **(C)** during one hospital stay by Abbott Freestyle Libre Flash Glucose Monitoring System. TIR, time in range. TBR, time below range. TAR, time above range. **(D)** Body weight and HbA1c levels after suffering from hypoglycaemia. **(E)** Blood glucose and serum insulin levels during overnight fasting and refeeding monitoring.

The patient was a 27-year-old Chinese woman with no family history of diabetes. Insulin therapy was initiated after she was diagnosed with T1D at 12 years of age. Since 2016, she has been hospitalized almost once a month with chief complaints of vomiting and abdominal pain. The onset of vomiting seemed to be associated with menstruation (**Figure 1A**), but attempts to create artificial menstrual cycles to prevent abdominal pain by taking oral contraceptives were failed and the levels of sexual hormones were in normal ranges during her menstrual cycle. Each time before the full-blown episode of vomiting, the patient had an impending sense of doom and came to our hospital for help in a state of panic. A few hours after admission, the patient started to have relentless nausea, vomiting, and retching, accompanied with abdominal pain. The episodes were so severe that the patient had vomiting episodes more than 30 times a day and the vomiting volume could be as large as 6 liters. Meanwhile, the patient was always in a manic mood due to the unbearable abdominal pain. During the period of vomiting, obvious activation of HPA axis was observed, evidenced by hypersecretion of adrenocorticotropic hormone and cortisol (**Table 1**). The blood glucose levels usually went up quickly and the insulin dosage had to be increased to avoid diabetic ketoacidosis (**Table 1**). Sometimes, both fever and elevated blood pressure and C-reactive protein were observed (**Table 1**). Usually 2-3 days after admission, the symptoms of vomiting and abdominal pain gradually improved and disappeared. After that, the patient started to experience fluctuating blood glucose with severe hypoglycaemia (**Figure 1B**), despite tight control of continuous subcutaneous insulin glulisine infusion (0.5U/h, 8-10; 0.25U/h, 10-12; 0.1U/h, 12-15; 0.05U/h, 15-24; 0.05U/h, 5-7) *via* smart insulin pump (Medtronic, Models MMT-712). 3-4 days later, the patient returned to basal condition without hypoglycaemia and was subsequently discharged (**Figure 1C**). At home, the patient had no symptoms of vomiting or abdominal pain and maintained stable glycaemia with daily infusion of total 3.2U insulin glulisine using insulin pump. The patient started to have severe hypoglycaemia in July of 2017, after that her HbA1c levels decreased from 10.9% to 5.9% (**Figure 1D**) and her body weight increased rapidly from 60.1 to 97.6kg (**Figure 1D**) and she developed obesity with a body mass index (BMI) of 31.7kg/m^2^.

**Table 1:** Laboratory measures for the vomiting period and hypoglycaemic period during one hospital stay.

Measure	Vomiting period	Hypoglycaemic period	Reference range
Body temperature (°C)	38.5 ↑	36.5	36.1-37.2
Blood pressure (mmHg)	145/90 ↑	117/78	90/60-120/80
ACTH (pg/mL)	175.2 ↑	11.7	7.2-63.6
Cortisol (µg/dL)	28.5 ↑	10.3	6.7-22.6
Blood glucose (mM)	20.15 ↑	1.74 ↓	4.11-6.05
Ketone bodies (mM)	1.7 ↑	0.1	<0.6
CRP (mg/L)	7.25 ↑	1.27	0-5
C-peptide (ng/mL)	<0.01 ↓	<0.01 ↓	1.1-4.4
Endogenous insulin (µU/mL)^*^	<0.2 ↓	<0.2 ↓	2.6-24.9
Exogenous insulin (µU/mL)^#^	13.4	27.0	–
Insulin antibodies (%)	0.34	21.83 ↑	0.00-5.00
Insulin receptor autoantibodies	Negative	Negative	Negative
IGF-1 (µg/L)	152	168	116-358
IGF-2 (µg/L)	143	125	100-200
GH (ng/mL)	0.232	0.224	0.010-3.607
GADA (U/mL)	19384 ↑	20760 ↑	<5

ACTH, adrenocorticotropic hormone; CRP, C-reactive protein; GADA, glutamic acid decarboxylase autoantibody; GH, growth hormone; IGF, insulin-like growth factor; ↑, higher than normal; ↓, lower than normal. *, measured by Roche Elecsys Cobas E601 Analyzer. #, measured by in-house prepared ELISA kit (#31380, ImmunoDiagnostics).

In order to clarify the pathogenic factors causing recurrent vomiting and abdominal pain, intensive whole-body physical examination and imaging diagnosis were performed, including ultrasonography, endoscopy, CT and MRI scan. But no significant findings were observed. Gastric emptying tests were normal. Whole blood cell counts as well as urine and stool examination were normal. Surprisingly, extremely high titer of glutamic acid decarboxylase autoantibody (GADA) was identified in the patient’s plasma despite it had almost been 12 years since the onset of T1D (**Table 1**). According to the Rome IV criteria (6), the patient was finally diagnosed with CVS. Supportive treatments with behavioral modification therapy were adopted to alleviate the symptoms of vomiting and avoid any probable triggers in our patient, but they were not effective in reducing the vomiting episodes.

It was unclear as to why the patient experienced spontaneous hypoglycaemia after having recovered from paroxysms of vomiting. Factitious hypoglycaemia was excluded in an insulin-inaccessible environment. During a hypoglycaemic attack, the endogenous insulin and C-peptide were undetectable, while the injected exogenous insulin was abundant in the blood sample (**Table 1**). To make clear of the hypoglycaemic excursion, the patient was intravenously injected with one dose of 0.4U insulin Lispro at 7pm before meal and blood glucose was monitored subsequently without any medical interference. A delayed fasting hypoglycaemia of 2.80mM blood glucose happened at 2:00am on the next early morning, which was followed with a slow elevation of blood glucose to 14.19mM before breakfast at 7:00am (**Figure 1E**). After breakfast, the blood glucose rapidly decreased to 3.66mM at 10:00am, which was then followed with a reactive hyperglycaemia of 12.12mM blood glucose at 11:00am (**Figure 1E**). These data suggested this patient had both fasting and postprandial hypoglycaemia alternating with reactive hyperglycaemia. Insulinoma was excluded due to normal pancreas morphology and negative tumor biomarkers. Meanwhile, other factors potentially causing hypoglycaemia, including insulin growth factors (IGFs) and IRAb, were normal or negative (**Table 1**). A positive response to glucagon stimulation at the time of hypoglycaemia is indicative of insulin-mediated hypoglycaemia (**Figure S1**). At last, abundant IAs were identified in the patient’s plasma during hypoglycaemia (**Table 1**).

**Figure 2:**
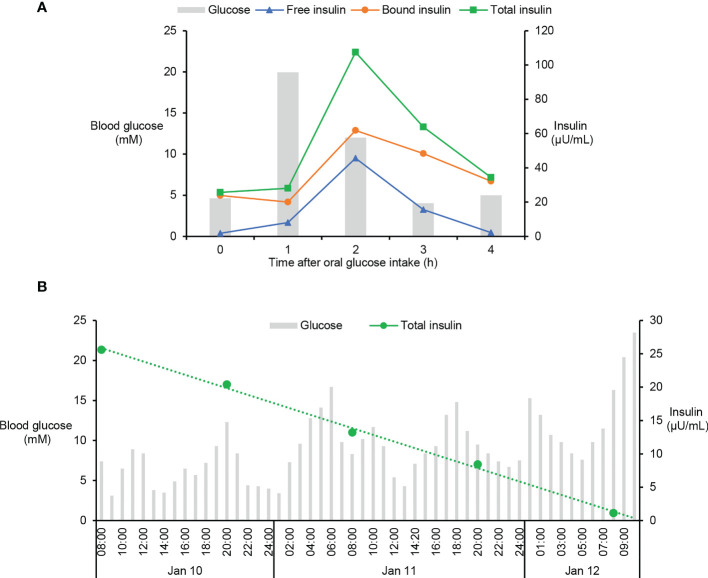
Fluctuation of blood glucose and serum insulin levels in oral glucose tolerance test and insulin withdraw study. **(A)** Oral glucose tolerance test was performed in this patient by oral ingestion of 75g of glucose preceded by an overnight fast and levels of blood glucose and serum insulin were measured at 0, 1, 2, 3, and 4h after glucose ingestion. Levels of free and total insulin were measured using the methods as described in **Figure S2**. Bound insulin was calculated by subtracting free insulin from total insulin. **(B)** After careful depletion of subcutaneous insulin, the patient was stabilized on intravenous insulin which was then discontinued. Blood glucose and serum insulin were monitored in the following time until the patient had sustained extreme hyperglycaemia, the sign of exhausted injected insulin. Total insulin was measured using the method as described in **Figure S2**.

Although it is extremely rare, antibodies against both endogenous and exogenous insulin are capable to induce intractable hypoglycaemia *via* binding with insulin and disrupting its normal function (14). Since the patient had high titers of IAs as well as undetectable C-peptide and endogenous insulin (**Table 1**), we speculated that the spontaneous hypoglycaemia in our patient was possibly caused by dysfunctions of injected insulin due to the presence of IAs. To test this hypothesis, both free and bound insulin levels were serially measured in the patient using methods developed and validated in house (**Figure S2**). Unexpectedly, both free and bound insulin levels simultaneously fluctuated with blood glucose in this patient (**Figure 1E**). At the hypoglycaemic points (2:00am, 10:00am), the free insulin levels were as low as 1.92 and 2.84µU/mL. At the hyperglycaemic points (7:00, 11:00am), the serum free insulin levels were as high as 9.50 and 24.57µU/mL. Meanwhile, the bound insulin levels showed similar trends as with free insulin levels. To further confirm this finding, we measured the patient’s insulin levels during an oral glucose tolerance test and found both the free and bound insulin levels also fluctuated along with the blood glucose levels (**Figure 2A**). Since the patient could not secrete any endogenous insulin and did not receive any exogenous insulin during the monitoring period, we revealed an unexpected phenomenon that the injected insulin was recycled in this patient with the fluctuation of blood glucose under the effects of IAs.

Considering the injected fast-acting insulin was not degraded quickly, but recycled in the blood of the patient (**Figures 1E**, **2A**), the half-life of the exogenous insulin must be prolonged by the effects of IAs. To estimate the half-life of injected insulin Lispro, an insulin withdraw study was performed in this patient. After careful depletion of subcutaneous insulin, the patient was stabilized on intravenous insulin which was then discontinued. Blood glucose and serum insulin were monitored in the following time until the patient had sustained extreme hyperglycaemia, the sign of exhausted injected insulin. Serial determinations of total insulin suggested that the circulating insulin had a prolonged serum half-time of 25h (**Figure 2B**). Since the patient had long history of T1D and undetectable C-peptide and endogenous insulin for many years, we concluded that the IAs were generated due to abnormal immune response to exogenous insulin and the patient was diagnosed with EIAS (13).

Several treatments were tried to inhibit the generation of IAs and the occurrence of hypoglycaemia in this patient, including changing insulin types, glucocorticoid treatment, intravenous immunoglobulin therapy (32.5g once a day by intravenous injection) and plasmapheresis (total 5 times one day apart). However, all these treatments could only temporarily prevent the hypoglycaemic episodes from relapsing. Finally, treatment with one cycle of rituximab consisted of two doses 2 weeks apart administered at the dose of 750mg/m^2^ body surface area was associated with a striking amelioration of hypoglycaemia, accompanied with significantly reduced titers of IAs (from 25% to 4%). Unexpectedly, the symptoms of cyclic vomiting were also remarkably alleviated during the follow up for 8 month.

## Discussion

CVS consists of four phases. Phase I is the asymptomatic interval between the vomiting episodes, which is followed by prodromal phase (phase II) with nausea and indisposition and the emetic phase (phase III), characterized by intense nausea, vomiting and often additionally abdominal pain. The vomiting episodes are mostly stereotypic with a mean of 6-12 times per year and last some hours to 7 days. Once vomiting attacks stop, recovery period (phase IV) begins and lasts minutes to some days. After phase IV, the CVS proceeds again to symptom-free interval (phase I) (6, 15). The cyclical patterns of vomiting in our case are highly stereotyped in respect of their hours of onset, symptomatology, frequency, and length and consistent with the four phases of CVS. Therefore, the patient was considered to have met all of the diagnostic criteria of CVS (7). Notably, menstruation seemed to be a trigger of her vomiting symptom, which is consistent with previous reported cases (16). However, we also believe it was necessary to exclude diabetic gastroparesis (DG) from the differential diagnosis, although DG presenting with similar cyclical patterns as CVS is uncommon (17). Compared with age- and sex-matched nondiabetic population, patients with T1D have over 30-fold risks of developing gastroparesis (18), which is characterized by delayed gastric emptying and moderate to severe upper gastrointestinal symptoms, including early satiety, postprandial fullness, nausea, vomiting, bloating, upper abdominal pain, and weight loss (17). Considering our patient had normal gastric emptying without weight loss, DG is not possible.

CVS is a rare disease, which could occur in all age groups with a prevalence of approximately 2% in childhood and less frequent in adults. Adults typically develop CVS in middle age with a female predominance (19). The exact pathogenesis and etiology of CVS are still not clear and there is no specific test to confirm a CVS diagnosis (20). Our patient with multiple kinds of autoimmune diseases and autoantibodies showed remission of vomiting symptoms after rituximab therapy, indicating CVS may be an underlying autoimmune disorder. To test this hypothesis, the prevalence of co-existence of autoimmune diseases or autoantibodies with CVS should be investigated in the future. Up to date, there are no evidence-based targeted treatments of CVS. The emetic phase of CVS is debilitating and usually requires medical attention. Identification and avoidance of precipitating factors in daily life are effective measures to prevent CVS. Antidepressant, antiepileptic, and antimigraine medications show an overall reduction or remission of CVS symptoms in more than 70% of patients (20). Much more is needed to really understand the etiology, pathophysiology, and pharmacotherapy of CVS.

Although the production of IAs has been decreased due to the wide use of hypoallergenic recombinant human insulin, it was reported that the prevalence of IAs is about 40% in insulin users administered human insulin and insulin analog formulations (21). It remains controversial whether IAs caused by exogenous insulin injection have significant effects on insulin efficacy or glycaemic control (22–24). Some rare cases show IAs may cause symptoms of extreme hyperglycaemia, frequent reoccurrence of hypoglycemia or both, which is termed EIAS. Our literature review identified 50 cases of EIAS with median age of symptom presentation of 70 years (range 15~85 years) and a slightly male predominance (M 68%; F 32%) (**Table 2**). 68% of the patients had intractable hypoglycaemia. Both patients with T1D or T2D might have EIAS under insulin therapy. Overall, it seems all the types of insulin can induce the generation of IAs and therefore cause EIAS from these case reports, including NPH insulin, regular insulin, NovoRapid, NovoMix, Humalog and etc. A recent epidemiological study revealed that the proportion of positive IAs was lowest in patients using glargine only and patients using glucose-lowering drugs (sulfonylureas/glinides, metformin, and DPP-4 inhibitors) had lower IAs levels than patients without these drugs, suggesting insulin glargine and the combination of oral glucose-lowering drugs may be useful to reduce IAs (46). The mechanisms underlying insulin antibodies production with recombinant human insulin injection are unknown. Factors that can lead people with diabetes to produce IAs include the recipient’s immune response genes, age, the insulin purity, molecular structure, storage condition, formulation of insulin and the sites and methods of insulin delivery (47). The dysglycaemic symptoms are usually alleviated by changing insulin formulations or discontinuing the insulin and switching to oral antidiabetic agents, but some patients are resistant to these treatments and other aggressive approaches have to be tried with varying success, including high-dose glucocorticoids, plasmapheresis, and rituximab (36). In our case, we have tried all the available therapeutic strategies and finally found rituximab was effective in lowering the IAs titer and correcting the recurrent hypoglycaemic symptoms.

**Table 2:** Literature review of diabetic cases with exogenous insulin antibody syndrome.

Year	Ref	n	Country	Sex	Age	Diabetes type	Diabetes duration (year)	HbA1c (%)	Hypoglycaemia	Insulin type	Clinical course
1984	(12)	1	US	F	28	1	13	–	Y	NPH insulin, Regular insulin	Uncurable by adjustments of insulin dose, type, or injection route
1997	(25)	1	Finland	M	27	1	3	7.3	Y	NPH insulin, Regular insulin	Reduced IAs and HbA1c levels by Lispro
1997	(26)	1	Japan	M	74	2	32	–	Y	Humulin N/R	Resolution of hypoglycaemia by prednisone and acarbose
2003	(27)	1	Japan	F	54	2	21	9.1	N	NPH insulin	Reduced IAs and HbA1c levels by Lispro
2004	(28)	1	Korea	F	72	2	0.06	6.3	Y	–	Resolution of hypoglycaemia by prednisone and glucose tablets
2005	(29)	1	Japan	M	73	1	31	–	Y	NPH insulin, Regular insulin	Euglycaemia obtained by using lispro insulin after 3 sessions of double filtration plasmapheresis and subsequent prednisolone treatment
2006	(30)	1	Japan	M	75	1	22	10	Y	Penfill N/R/30R	Glycaemic control was improved by prednisolone and methylprednisolone
2009	(31)	2	Japan	M	86	–	20	7.2	Y	–	Resolution of hypoglycaemia by prednisolone and cyclophosphamide
				M	83	–	45	10.1	Y	Lispro	Resolution of hypoglycaemia by double filtration plasmapheresis, prednisolone and cyclophosphamide
2010	(32)	1	Korea	F	71	2	3	8.7	Y	Humulin N	Resolution of DKA and hypoglycaemia by acarbose and prednisolone
2010	(33)	1	China	M	82	2	5	8.6	Y	Novolin 30R	Glycaemic control was improved by acarbose and metformin
2011	(34)	1	Japan	M	70	2	35	11.9	Y	Lispro, Detemir	Glycaemic control was improved by glulisine
2014	(35)	1	UK	F	15	1	1	7.5	N	–	Stable condition obtained after treatment of rituximab and methylprednisolone
2015	(36)	11	China	F	77	1	0.42	5.6	Y	NovoMix 30	Reduced hypoglycaemia frequency by glinides
				M	55	1	3	7.3	Y	NovoRapid	Remission of hypoglycaemia by lifestyle modification
				F	46	1	0.67	6.1	Y	NovoMix 30	Remission of hypoglycaemia by acarbose
				F	77	1	4	5.7	Y	NovoMix 30	Remission of hypoglycaemia by acarbose
				M	62	1	2	5.9	Y	NovoRapid +Lantus	Remission of hypoglycaemia by acarbose
				M	71	1	17	6.5	Y	Humalog, Mix25	Remission of hypoglycaemia by acarbose
				M	65	1	8	6.4	Y	NovoRapid	Remission of hypoglycaemia by acarbose
				M	79	1	10	9.6	Y	NovoRapid	Glycaemic control improved by nateglinide, acarbose and januvia
				M	70	1	1	6.3	Y	Humalog, Mix25	Remission of hypoglycaemia by lifestyle modification and received glucocorticoid therapy
				M	61	1	6	6.8	Y	Wan Sulin 30R#	Remission of hypoglycaemia by RHII
				M	82	1	15	8.6	Y	Novolin 30R	Remission of hypoglycaemia by acarbose and biguanide
2015	(21)	12	China	M	76	2	11	10.7	Y	MPZRHII70/30	Reduced IAs and HbA1c levels by glargine, aspart and acarbose
				M	72	2	15	9.0	Y	MPZRHII70/30	Reduced IAs and HbA1c levels by lispro and glargine
				F	62	2	13	10.5	Y	MPZRHII70/30	Reduced IAs and HbA1c levels by repaglinide and pioglitazone
				M	70	2	19	9.5	Y	IPBHII30R	Reduced IAs and HbA1c levels by lispro, glargine and prednisone
				M	67	2	13	9.1	N	MPZRHII70/30	Reduced IAs and HbA1c levels by lispro 25
				F	75	2	15	10.2	N	MPZRHII70/30	Reduced IAs and HbA1c levels by lispro 25 and acarbose
				F	68	2	17	9.7	N	MPZRHII70/30	Reduced IAs and HbA1c levels by acarbose and nateglinide
				M	80	2	9	10.1	N	IPBHII + BHII	Reduced IAs and HbA1c levels by glargine and Acarbose
				F	78	2	27	12.2	N	IPBHII50R	Reduced IAs and HbA1c levels by acarbose and metformin
				F	68	2	19	11.4	N	IPBHII30R	Reduced IAs and HbA1c levels by aspart 30, acarbose and metformin
				F	58	2	13	10.3	N	IPBHII + BHII	Reduced IAs and HbA1c levels by glargine, acarbose and rosiglitazone
				F	56	2	12	9.3	N	IPBHII50R	Reduced IAs and HbA1c levels by aspart and glargine
2016	(37)	1	Taiwan	M	48	1	20	7.6	Y	Aspart, Glargine, Humulin N/R	Hypoglycaemia was relieved after prednisolone treatment
2016	(38)	1	China	M	64	2	10	6.1	Y	Aspart 30	No recurrence of hypoglycaemia after dietary and behavioral interventions
2017	(39)	2	China	M	62	2	1	–	Y	PZRHII	No recurrence of hypoglycaemia by metformin and sitagliptin
				M	83	2	32	8	Y	RHII, PZRHII	No recurrence of hypoglycaemia by nateglinide and acarbose
2017	(40)	1	Japan	M	62	1	0.06	8.3	N	Lispro, Detemir	Glycaemic control was improved by prednisolone and double filtration plasmapheresis
2018	(41)	2	China	M	76	2	20	5.8	Y	Aspart 30	No recurrence of hypoglycaemia by prednisone
				M	50	2	10	6.5	Y	Aspart 30R	No recurrence of hypoglycaemia by prednisone
2019	(42)	4	China	M	79	2	14	10.2	Y	Lispro, Humulin R	Glycaemia returned to normal range by acarbose and sitagliptin
				M	71	2	11	–	Y	Aspart 30	Glycaemic control was improved by acarbose, metformin and glargine
				M	79	2	4	–	Y	Aspart 30	Glycaemic control was improved by human biosynthetic insulin
				M	52	2	3	–	Y	Gansulin 50R	Resolution of hypoglycaemia by metformin, acarbose, aspart, and methylprednisolone
2020	(43)	1	US	M	32	1	5	–	Y	Glargine, Lispro	Glycaemic control was improved by IVIG
2021	(44)	1	China	M	43	2	23	–	N	Glulisine, Aspart, Glargine	Endogenous insulin increased by TPE and IVIG
2021	(45)	1	US	F	51	1	0.04	11.8	Y	Detemir, Aspart	Reduced IAs and HbA1c levels by CSII and mycophenolate

M, Male; F, Female; N, No; Y, Yes; BHII, biosynthetic human insulin injection; CSII, continuous subcutaneous insulin infuse; DKA, diabetic ketoacidosis; NPH, neutral protamine hagedorn; IPBHII, isophane protamine biosynthetic human insulin injection; IVIG, intravenous immune globulin; MPZRHII, mixed protamine zinc recombinant human insulin injection; PZRHII, protamine zinc recombinant human insulin injection; RHII, recombinant human insulin injection, TPE, therapeutic plasma exchange.

As for the mechanisms whereby how IAs cause dysglycaemia, there is a ‘reservoir-like effect’ hypothesis (13). IAs are able to first bind the insulin in circulation and therefore disrupt the normal function of insulin and cause hyperglycaemia by serving as a carrier. Later, the IAs may dissociate from the insulin, allowing the activation of cellular insulin receptors and leading to unexpected hypoglycaemia. However, this hypothesis cannot explain the spontaneous and intermittent hypoglycaemia symptoms in our patient who had no endogenous insulin production and did not receive exogenous insulin during the monitoring. We found the injected insulin was not degraded but recycled back to the circulation after the symptom of hypoglycemia in our patient. Therefore, we wonder whether the presence of IAs inhibits the degradation of insulin and therefore prolong the pharmacodynamic action of insulin. We noted that the patient experienced continuous weight gain after having IAs and hypoglycaemia, which is possibly caused by the anabolic effects of insulin. A previous case study showed that the insulin that was bound to low-affinity IAs still maintained biologic activity *in vivo* (12). It is possible that the IAs, insulin and insulin receptor can generate a cross-linking structure and therefore prevent insulin endocytosis mediated by insulin receptor on cell surface. Alternatively, IAs may facilitate insulin to enter recycling endosome and reduce insulin degradation in lysosome by binding with neonatal Fc receptor (**Figure S3**) (48). More molecular studies are needed to test these hypotheses. Elaborating the molecular mechanisms underlying IAs-induced dysglycaemia will contribute to the development of targeted therapeutics for EIAS.

We here report the first patient with concurrent CVS and EIAS in the setting of T1D. A series of methods for detection of endogenous, exogenous, free, bound or total insulin were tried and validated in house. Our case also stresses again the need to test IAs in patients presenting with intractable hypoglycaemia under insulin therapy. Importantly, our data provided a novel mechanistic insight into the dysglycaemia caused by IAs *via* extending the half-life of insulin.

## Data availability statement

The original contributions presented in the study are included in the article/**Supplementary Material**. Further inquiries can be directed to the corresponding authors.

## Ethics statement

The studies involving human participants were reviewed and approved by Institutional Review Boards of The University of Hong Kong-Shenzhen Hospital. The patients/participants provided their written informed consent to participate in this study. Written informed consent was obtained from the individual(s) for the publication of any potentially identifiable images or data included in this article.

## Author contributions

All authors contributed to the study conception and design. Material preparation, *in vitro* experiments, data collection and analysis were performed by LG, XD, HH, and YL. The first draft of the manuscript was written by LG, and all authors commented on previous versions of the manuscript. All authors contributed to the article and approved the submitted version.

## Funding

This project was supported by the Area of Excellence (AOE/M/707-18) from the Research Grant Council of Hong Kong as well as the National Natural Science Foundation of China (82070860 and 32000816).

## Conflict of interest

The authors declare that the research was conducted in the absence of any commercial or financial relationships that could be construed as a potential conflict of interest.

## Publisher’s note

All claims expressed in this article are solely those of the authors and do not necessarily represent those of their affiliated organizations, or those of the publisher, the editors and the reviewers. Any product that may be evaluated in this article, or claim that may be made by its manufacturer, is not guaranteed or endorsed by the publisher.
