# Supplementary material for: Type 1 diabetes complicated with cyclic vomiting syndrome and exogenous insulin antibody syndrome: A case report
Source: Front Endocrinol (Lausanne). 2022 Nov 10;13:1043301. doi: 10.3389/fendo.2022.1043301 (PMC9684460; doi:10.3389/fendo.2022.1043301)
Supplement: Supplementary file 1 [file DataSheet_1.pdf]

Supplementary Figure 1

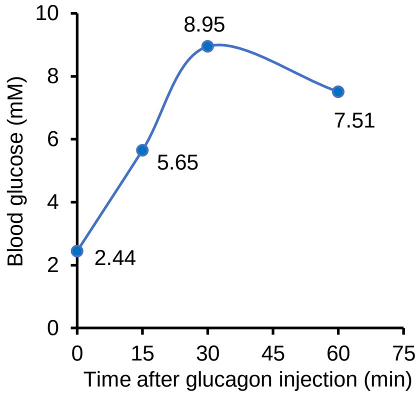

Figure S1. Glucagon stimulation test at the time of low blood glucose. The patient was injected with 1mg of glucagon when the patient had one episode of hypoglycaemia at the early morning, followed by blood glucose monitoring at 0, 15, 30, and 60min.

Supplementary Figure 2

A

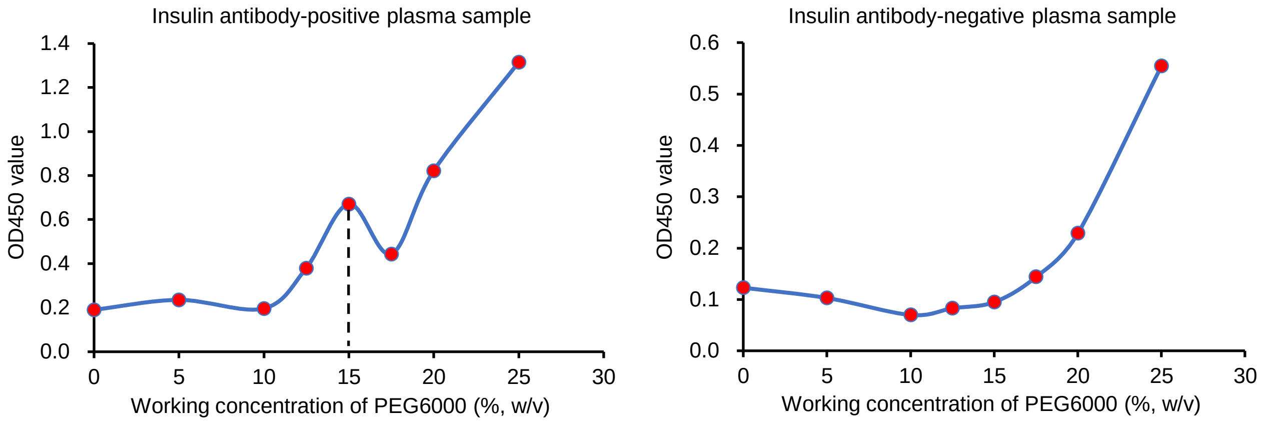

B

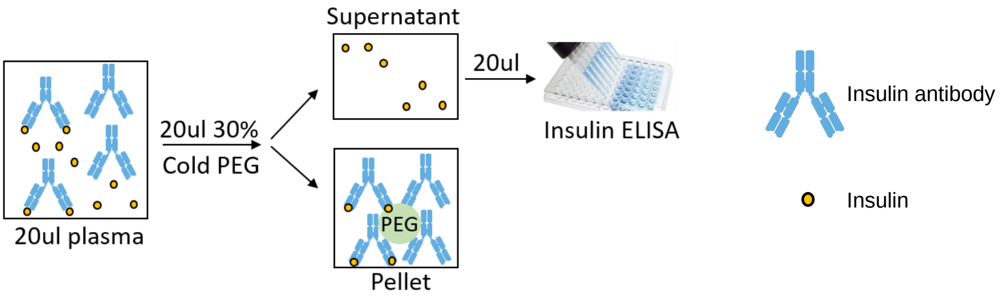

C

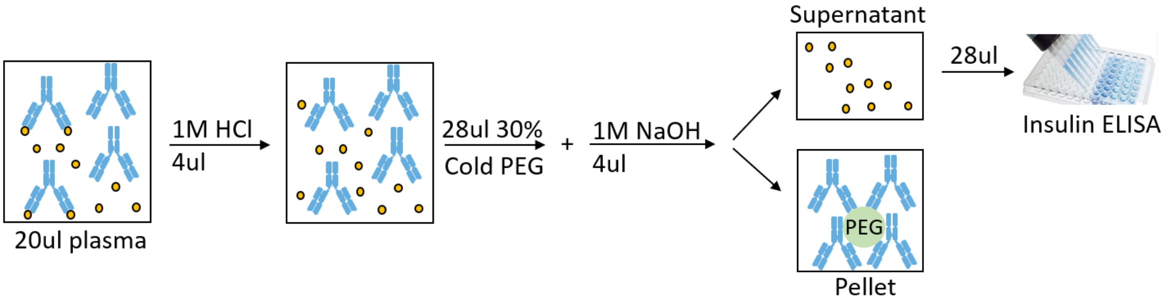

Figure S2. Detection methods for free and total insulin in plasma samples with insulin antibodies by PEG6000 precipitation.
(A) Plasma samples with insulin antibodies (left panel) or without insulin antibodies (right panel) were pre-treated with PEG6000 (#81255, Fluka Analytical) at different working concentrations (0, 5, 10, 12.5, 15, 17.5, 20, 25%, w/v). After mixing and centrifugation, the supernatants were subjected to insulin ELISA assays and the OD450 values were obtained. According to the OD450 values measured in insulin antibody-positive plasma samples, the optimal working concentration of PEG6000 to precipitate insulin antibodies should be 15% (w/v). If the working concentration of PEG6000 is lower than 15%, insulin antibodies can not be completely precipitated and removed, which causes lower results than the real values. If the working concentration of PEG6000 is 17.5%, other than insulin antibodies, insulin in the plasma sample will also be precipitated by PEG and lost, leading to lower measurements compared with the actual values. If the working concentration of PEG is more than 17.5%, excessive PEG in the supernatant will bind with coated protein in ELISA plate and capture detection antibody, causing false positive signals for both insulin antibody-positive and -negative plasma samples.
(B) The schematic diagram shows the principle for free insulin measurement in plasma samples with insulin antibodies. Plasma is mixed with equal volume of 30% cold PEG6000 (w/v) to make the working concentration of PEG6000 as 15% (w/v), followed by mixing and centrifugation. Free insulin in the supernatant is finally measured by insulin ELISA kit.
(C) The schematic diagram shows the principle for total insulin measurement in plasma samples with insulin antibodies. The bound insulin is firstly released from the insulin antibodies by acid pre-treatment. Then all the insulin antibodies are precipitated by PEG6000 and removed by centrifugation after alkali neutralization. The total insulin in supernatant is finally measured by insulin ELISA kit.

Supplementary Figure 3

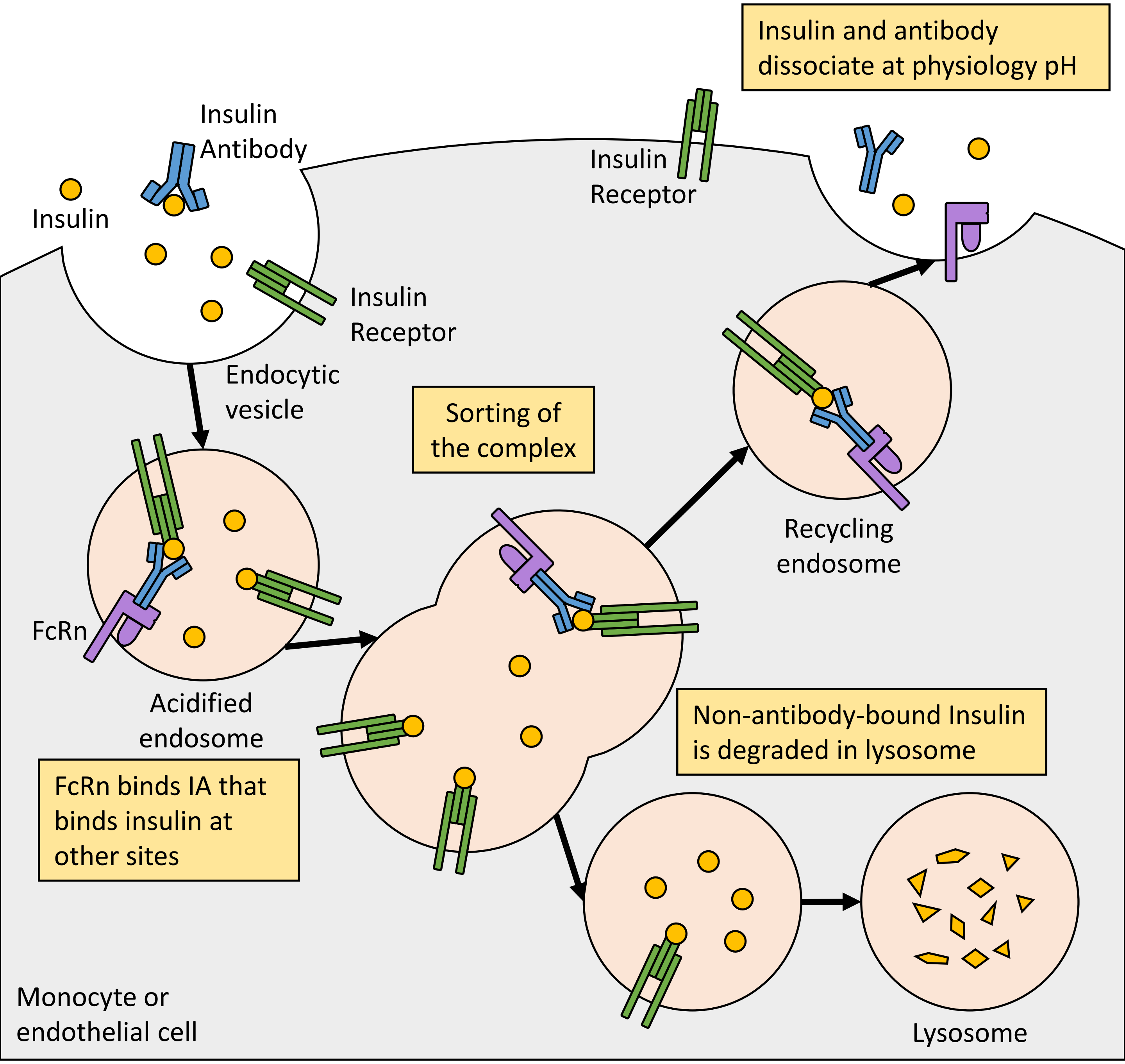

Figure S3. A hypothetic working model where insulin antibody inhibits insulin degradation via FcRn-mediated cellular recycling. Insulin antibody-insulin complex is internalized by binding with insulin receptor on the cell surface. The neonatal Fc receptor (FcRn) binds to insulin antibody-insulin complex in an acidic endosomal compartment. FcRn then recycles insulin antibody-insulin complex back into circulation, thus extending the serum half-life of insulin. Serum insulin without a recycling receptor is destined for lysosomal degradation.
